# Repression of nuclear CELF activity can rescue CELF-regulated alternative splicing defects in skeletal muscle models of myotonic dystrophy

**DOI:** 10.1371/currents.RRN1305

**Published:** 2012-03-13

**Authors:** Dara S. Berger, Andrea N. Ladd

**Affiliations:** Department of Cell Biology, Lerner Research Institute, Cleveland Clinic, 9500 Euclid Ave. NC10, Cleveland, OH 44195 USA

## Abstract

Myotonic dystrophy type 1 (DM1) is caused by the expansion of CUG repeats in the 3’ UTR of DMPK transcripts. DM1 pathogenesis has been attributed in part to alternative splicing dysregulation via elevation of CUG-BP, Elav-like family member 1 (CELF1). Several therapeutic approaches have been tested in cells and mice, but no previous studies had specifically targeted CELF1. Here, we show that repressing CELF activity rescues CELF-dependent alternative splicing in cell culture and transgenic mouse models of DM1. CELF-independent splicing, however, remained dysregulated. These data highlight both the potential and limitations of targeting CELF1 for the treatment of DM1.

## INTRODUCTION

Myotonic dystrophy (*dystrophia myotonica*, DM) is the most common form of adult-onset muscular dystrophy [1]. It is an autosomal dominant disease caused by expansion of CTG (DM1) or CCTG (DM2) repeats in the 3' UTR of the *DMPK* gene or intron 1 of the *ZNF9* gene, respectively [2] [3] [4] [5]. DM is a multi-system disease characterized by myotonia, muscle wasting, insulin resistance, cardiac conduction defects, cataracts, testicular atrophy, and cognitive dysfunction [1]. Congenital forms of DM are even more severe, exhibiting deficiencies in muscle formation, including poor fiber type differentiation [1]. The mechanism of pathogenesis is not fully understood, but several lines of evidence indicate that expanded CUG or CCUG repeat-containing RNA disrupts normal processing of other mRNAs [6]. These effects are mediated at least in part via disruption of the alternative splicing factor CUG-BP, Elav-like family member 1 (CELF1) [7] [8] [9] [10] [11]. 

CELF1 protein is normally down-regulated in striated muscle after birth [12] [13]. The postnatal down-regulation of CELF1 is post-transcriptional, and involves both changes in CELF1 phosphorylation that affect protein stability and microRNA-mediated repression of CELF1 protein production [14] [15]. In heart and skeletal muscle of adult DM1 patients, however, CELF1 protein levels are elevated [8] [16]. Activation of protein kinase C (PKC) in DM1 by an as yet unknown mechanism leads to the hyperphosphorylation and stabilization of CELF1 protein [15]. CELF1 is also up-regulated in heart and skeletal muscle in transgenic mice following induction of expanded CUG repeat RNA expression [17] [18]. Interestingly, transgenic mice that constitutively over-express CELF1 in skeletal muscle share many of the same pathological features of congenital DM1 patients [10] [19], while mice that are induced to over-express CELF1 in adult skeletal muscle develop many of the features of adult-onset DM1, including muscle wasting [20]. 

The dysregulation of the alternative splicing of CELF targets in DM1 has been directly linked to skeletal muscle symptoms. Alternative splicing of insulin receptor (*IR*) contributes to insulin resistance [8], whereas alternative splicing of a muscle-specific chloride channel (*ClC-1*) leads to myotonia [9] [21] [22] [23]. Splicing patterns in adult DM1 patient muscle tissues are consistent with elevated CELF activity, and resemble normal fetal splicing patterns despite a lack of extensive regeneration in DM1 muscle [8] [24]. This suggests that pathogenesis is due to aberrant regulation of alternative splicing that leads to an inappropriate reiteration of the fetal CELF splicing program in adult. 

Up-regulation of CELF1 is not the only mechanism by which alternative splicing is dysregulated in DM1. Another alternative splicing regulator, muscleblind-like 1 (MBNL1) co-localizes with expanded CUG repeat RNA [25] [26], and it has been proposed sequestration of MBNL1 into expanded repeat-containing RNA foci leads to loss of MBNL1 function. Skeletal muscles from mice lacking MBNL1 do exhibit changes in alternative splicing, histology, and function that are characteristic of DM1, though they do not develop a full DM1 phenotype [11] [27] [28] [29] [30] [31]. Taken all together, the body of evidence indicates that up-regulation of CELF1 and loss of MBNL1 function both play important roles in DM1 pathogenesis. 

Based on what is known about the causative factors that contribute to DM1 pathogenesis, a number of therapeutic approaches have been tested in cultured cells and mouse models with encouraging results. Morpholino antisense oligonucleotides targeting a splice site in *ClC-1* was able to reverse the *ClC-1 *alternative splicing defect in skeletal muscle and eliminate myotonia in two mouse models of DM1 [23]. Approaches to restore the loss of MBNL1 function in skeletal muscle have included over-expression of MBNL1 protein via a recombinant adeno-associated viral vector [32], and the administration of either a drug or morpholino antisense oligonucleotide to disrupt the interaction of MBNL1 with expanded CUG repeat-containing RNA [33] [34]. In each case, an increase in MBNL1 function at least partially rescued alternative splicing defects in mice. Administration of a PKC inhibitor reduced CELF1 protein levels, rescued alternative splicing of some transcripts, and improved cardiac function in an inducible heart-specific mouse model of DM1 [35]. Finally, oligonucleotides or peptides targeted against the expanded CUG repeat-containing RNA reduced foci formation and alternative splicing dysregulation in a DM1 mouse model [36] [37]. 

Despite the evidence that an increase in CELF1 protein contributes to DM1 pathogenesis, to date no one has directly targeted CELF1 in DM1 muscle. In this study, we tested whether repression of CELF activity in skeletal muscle would restore normal alternative splicing patterns in cultured cells and a mouse model of DM1. We demonstrate that expression of a dominant negative CELF protein, NLSCELF∆, is sufficient to reverse dysregulation of a splicing minigene by expanded CUG repeat-containing RNA in differentiated C2C12 cells. In mice, NLSCELF∆ was also able to rescue dysregulation of alternative splicing of a CELF target RNA, but had no effect on a CELF1-independent transcript. These data highlight the potential and limitations of CELF1 as a therapeutic target for the treatment of DM1. 

## MATERIALS AND METHODS 

### Co-transfection experiments 

 All plasmids have been previously described. The R35C minigene is a chicken cTNT exon 5 alternative splicing reporter [38]. DMPKS expresses a partial DMPK transcript containing exons 11-15 without expanded CUG repeats [7], whereas DT960 expresses the same DMPK transcript with 960 CUG repeats in exon 15 [25]. The NLSCELF∆ construct expresses a nuclear dominant negative CELF protein with an Xpress epitope tag [39]. 

C2C12 murine myoblasts (ATCC) were maintained in growth medium (low-glucose DMEM supplemented with 20% FBS, 0.5% chick embryo extract, and 1% antibiotic/antimycotic). C2C12 cells were transfected with 100 ng R35C, 0 or 250 ng DMPKS or DT960, 0 or 2 µg NLSCELF∆, and carrier plasmid as needed to a total of 3.1 µg DNA per 60 mm plate at 30-40% confluency (d_-1_) using Fugene 6 (Roche Applied Science). Two days later when cells had reached 95-100% confluency (d_1_), medium was changed to differentiation medium (low-glucose DMEM supplemented with 5% horse serum, and 1% antibiotic/antimycotic). Medium was replaced with fresh differentiation medium the following day (d_2_), and samples were collected the day after that (d_3_). By d_3_, C2C12 cells had differentiated into large, multi-nucleated myotubes. Total protein and RNA samples were harvested from parallel plates. Protein samples were collected and NLSCELF∆ protein expression was confirmed by western blotting as previously described [38]. RNA was harvested using Trizol reagent (Invitrogen), and the extent of minigene exon inclusion was determined by semi-quantitative RT-PCR as previously described [38]. 

### Bitransgenic mice 

Animal husbandry and experiments were performed under the approval of the Cleveland Clinic Institutional Animal Care and Use Committee (Protocol number ARC 08612) and in accordance with the recommendations of the American Veterinary Medical Association. 

HSA^LR^ line 20b transgenic mice [40], which are normally maintained as homozygotes in an FVB background, were crossed with FVB-N wild type mice (Taconic) to generate HSA^LR^-20b hemizygotes. Myo-CELF∆-370 transgenic mice [41], which are normally maintained as hemizygotes in a B6SJL hybrid background, were crossed into an FVB-N background for six generations to produce hemizygous Myo-CELF∆-370-FVBN6 mice. Myo-CELF∆-370-FVBN6 mice were crossed with HSA^LR^-20b hemizygotes to generate wild type, hemizygous HSA^LR^-20b (referred to in the Results simply as “HSA^LR^”), and hemizygous bitransgenic HSA^LR^-20b/Myo-CELF∆-370-FVBN6 (referred to in the Results as “HSA^LR^/Myo-CELF∆”) littermates. Thigh muscles were collected from sex- and age-matched adult mice. Total RNA was extracted using Trizol reagent (Invitrogen). Alternative splicing of *Nrap* exon 12 and *Serca1* exon 22 was evaluated by semi-quantitative RT-PCR as described by Lin and colleagues [11]. 

### Statistics 

 Comparisons between the mean values for the extent of exon inclusion and skipping in different experimental groups were performed via non-parametric, one-tailed Mann-Whitney *U*-test. Differences were considered statistically significant when P ≤ 0.05. 

### RESULTS 

#### Repression of CELF activity rescues alternative splicing of a minigene reporter in a C2C12 cell culture model of DM1 

Since an increase in CELF protein expression likely contributes to pathogenic changes in alternative splicing in skeletal muscle of DM1 patients, we reasoned that repression of CELF activity should restore normal alternative splicing patterns. It has been previously shown that cardiac troponin T exon 5 inclusion is elevated in DM1 [7], and co-expression of *cTNT* minigenes with RNAs containing expanded CUG repeats by transient transfection recapitulates this effect in both fibroblasts and skeletal muscle cells [7] [25]. Likewise, we observed that co-transfection of a *cTNT* minigene (R35C) with a plasmid expressing a partial *DMPK* transcript containing 960 CUG repeats (DT960) induced a modest but significant increase in exon inclusion compared to the minigene alone in C2C12 myoblasts that had been differentiated into myotubes (Figure 1). An identical *DMPK* transcript lacking the CUG repeats (DMPKS) had no significant affect on exon inclusion (data not shown).

**Figure:**
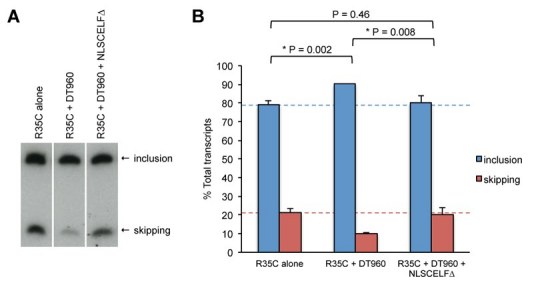
**Figure 1.  Alternative splicing changes induced by an expanded CUG repeat-containing RNA can be rescued in cultured cells by repression of CELF activity.**  Expression of an RNA containing 960 CUG repeats promotes exon inclusion of the R35C chicken *cTNT* minigene reporter in differentiated C2C12 myotubes.  This effect is rescued by additional co-expression with NLSCELF∆, a dominant negative CELF protein.  (**A**) A representative RT-PCR gel showing R35C alternative exon included and exon skipped products.  (**B**) Quantitation of the extent of R35C exon inclusion and exon skipping in multiple independent transfection experiments (n = 4 to 6 samples per group).

The R35C minigene has also been shown to respond to CELF-mediated regulation. CELF protein over-expression promotes exon inclusion in fibroblasts [38], reminiscent of the effects of the DT960 expanded CUG repeat-containing RNA. In contrast, repression of endogenous CELF activity in primary embryonic cardiomyocytes by expression of a dominant negative CELF protein, NLSCELF∆, promotes R35C exon skipping [13]. To determine whether repression of CELF activity would counteract the induction of exon inclusion induced by expanded CUG repeat-containing RNA in skeletal muscle cells, we co-transfected C2C12 cells with R35C, DT960, and NLSCELF∆ expression plasmids and induced myotube differentiation. Expression of the NLSCELF∆ protein was confirmed by western blotting (data not shown). Exon inclusion of the minigene following co-expression of both DT960 and NLSCELF∆ was similar to that of the minigene alone (Figure 1), suggesting that repression of CELF activity in the nucleus is sufficient to ameliorate the affects of expanded CUG repeat-containing RNA on alternative splicing of this transcript in skeletal muscle cells. 

#### Repression of CELF activity partially rescues alternative splicing in a DM1 mouse model *in vivo*

 Unfortunately, expression of an expanded CUG repeat-containing RNA by transient transfection was insufficient to disrupt alternative splicing of endogenous transcripts in C2C12 myotubes (data not shown), perhaps due to inadequate transfection efficiency in these cells. To extend these studies to endogenous skeletal muscle transcripts *in vivo*, we employed two pre-existing transgenic mouse models. The Thornton laboratory developed the HSA^LR^ mouse model of DM1, in which 250 untranslated CUG repeats are expressed in skeletal muscle under the human skeletal actin promoter [40]. Skeletal muscles in HSA^LR^ mice display alternative splicing changes associated with DM1, as well as myopathy and myotonia, common clinical manifestations of DM [11] [22] [40]. Our laboratory has generated Myo-CELF∆ transgenic mice in which CELF activity is specifically repressed in skeletal muscles by expression of the NLSCELF∆ dominant negative protein under the mouse myogenin promoter [41]. Myo-CELF∆ mice exhibit dysregulation of alternative splicing of some CELF targets in skeletal muscle, as well as changes in muscle fiber properties [41]. Myo-CELF∆ mice have reduced, not elevated, CELF activity, and therefore are not themselves a model of DM1. Many of the same muscle properties (i.e. interstitial spacing, fiber size variability, and fiber type composition) are affected, however, consistent with a role for CELF-mediated alternative splicing in regulating these characteristics of skeletal muscle pathogenesis in DM1 [41]. 

 To test whether repression of CELF activity would rescue alternative splicing defects in an *in vivo* model of DM1 skeletal muscle, we crossed HSA^LR^ mice to a line of Myo-CELF∆ mice to generate bitransgenic HSA^LR^/Myo-CELF∆ mice. Thigh muscle was collected from age- and sex-matched wild type, HSA^LR^ transgenic, and HSA^LR^/Myo-CELF∆ bitransgenic littermates, and the alternative splicing of *Nrap* exon 12 was evaluated by RT-PCR (Figure 2). This alternative splicing event was chosen because it is known to be dysregulated in DM1, and to be regulated by CELF proteins. *Nrap* exon 12 inclusion is reduced in skeletal muscles of DM1 patients and HSA^LR^ mice [11], but elevated in Myo-CELF∆ mice [41]. Consistent with previous reports [11], we saw a significant decrease in *Nrap* exon 12 inclusion in thigh muscle from hemizygous HSA^LR^ mice relative to their wild type littermates (Figure 2). In contrast, bitransgenic HSA^LR^/Myo-CELF∆ mice exhibited a level of inclusion that is significantly higher than that seen in HSA^LR^ mice, and that is similar to that of wild type. 

**Figure:**
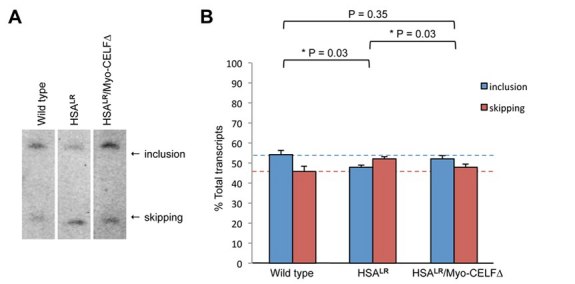
**Figure 2.  Alternative splicing of a known CELF target is rescued in** **bitransgenic** **HSA** **^LR^** **/** **Myo** **-CELF∆ mice.** *Nrap* exon 12 skipping is induced in the thigh muscle of expanded CUG repeat RNA-expressing HSA^LR^ mice, but is normal in bitransgenic HSA^LR^/Myo-CELF∆ transgenic mice that also express a dominant negative CELF protein.  (**A**) A representative RT-PCR gel showing *Nrap* exon 12 included and exon skipped products in thigh muscle total RNA samples from wild type, transgenic, and bitransgenic mice.  (**B**) Quantitation of the extent of exon inclusion and exon skipping in thigh muscle samples from multiple individuals (n = 6 for wild type, n = 5 for HSA^LR^, and n = 6 for HSA^LR^/Myo-CELF∆).

 Not all transcripts that are dysregulated in DM1 are sensitive to CELF regulation. For example, *Serca1* exon 22 skipping is observed in skeletal muscle of DM1 patients and HSA^LR^ mice, but not in healthy muscle [42]. Over-expression of CELF1 did not, however, affect the alternative splicing of a mouse *Serca1* minigene or endogenous *Serca1* transcripts in C2C12 cells [43]. MBNL1, on the other hand, is a positive regulator of *Serca1* exon 22 inclusion [43]. Together, these results suggest that the dysregulation of *Serca1* exon 22 alternative splicing in DM1 is due primarily to the disruption of MBNL1 activity, with no contribution from the up-regulation of CELF1. Consistent with this, the decrease in *Serca1 *exon 22 inclusion observed in HSA^LR^ mice was not rescued in HSA^LR^/Myo-CELF∆ bitransgenic animals (Figure 3). 

**Figure:**
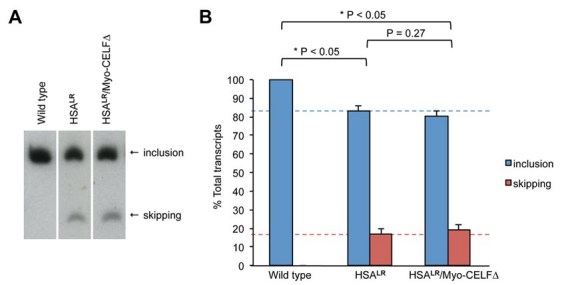
**Figure 3.  Alternative splicing of a non-CELF target RNA is not rescued in** **bitransgenic** **HSA** **^LR^** **/** **Myo** **-CELF∆ mice.** *Serca1* exon 22 is aberrantly skipped in the skeletal muscle of HSA^LR^ mice, which express an RNA containing 250 CUG repeats.  Aberrant *Serca1* exon 22 alternative splicing is not rescued by crossing HSA^LR^ mice with Myo-CELF∆ transgenic mice, which express a dominant negative CELF protein in skeletal muscle. (**A**) A representative RT-PCR gel showing *Serca1 *exon 22 included and exon skipped products in thigh muscle total RNA samples from wild type, transgenic, and bitransgenic mice.  (**B**) Quantitation of the extent of exon inclusion and exon skipping in thigh muscle samples from multiple individuals (n = 2 for wild type, n = 6 for HSA^LR^, and n = 5 for HSA^LR^/Myo-CELF∆).

## DISCUSSION 

There is currently no cure or specific treatment for DM1. Although one can argue that targeting the mutant expanded CUG repeat-containing RNA would be the most direct and comprehensive approach, we believe that until a safe and effective therapy has been validated in human DM1 patients alternative therapeutic targets should continue to be explored. In addition to the expanded CUG repeat-containing RNA, MBNL1 and PKC have shown some promise as therapeutic targets in cell culture and mouse models of DM1 [32] [33] [34] [35] [36] [37]. Although Wang and colleagues demonstrated that inhibition of PKC resulted in reduced CELF1 expression in a heart-specific mouse model of DM1 [35], this is the first study to specifically target CELF activity. Our data suggest that CELF1 is a viable therapeutic target, as repression of CELF activity restores normal alternative splicing of CELF-responsive dysregulated transcripts in cell culture and transgenic mouse models of DM1 skeletal muscle. 

Elevated CELF1 protein levels have been proposed to underlie muscle wasting, one of the more devastating clinical manifestations of DM1 [17]. The 250 CUG expanded repeat HSA^LR^ and MBNL1 knockout mouse models of DM1 show sequestration of MBNL1 but not up-regulation of CELF1, and exhibit only partial DM1 phenotypes with no muscle wasting [31] [40]. In contrast, the inducible 960 CUG expanded repeat EpA960/HSA-Cre-ERT2 (+tam) mouse model, which shows both sequestration of MBNL1 and elevated levels of CELF1 protein in skeletal muscle, has a more complete DM1 phenotype including muscle wasting [17]. In addition, fetal splicing patterns of *Ank2*, *Capzb*, and *Fxr1 *transcripts were seen in EpA960/HSA-Cre-ERT2 (+tam) mice and DM1 muscle tissue, but not in HSA^LR ^and MBNL1-null muscle [17]. Alternative splicing of these transcripts is regulated by CELF1, but not MBNL1, during normal heart development [12]. These studies indicate that there are alternative splicing events dysregulated in DM1 that can be attributed specifically to CELF1, and these may underlie some symptoms of the disease. 

 Several alternative splicing events that are dysregulated in DM1 respond to both elevation of CELF1 and loss of MBNL1, including *cTNT* [7] [38] [44], *IR* [8] [45] [46], *ClC-1* [9] [31], and *Nrap* [11] [41]. It is worth noting that although CELF1 is not elevated in HSA^LR^ mice, we found that repression of CELF activity was still sufficient to restore normal alternative splicing of *Nrap* exon 12 in this model (Figure 2). We did not observe restoration of normal *Serca1 *exon 22 alternative splicing, however, an alternative exon that is regulated by MBNL1 but not CELF1 [43]. Thus, just as therapies targeted specifically to MBNL1 would presumably not alleviate CELF1-dependent symptoms, neither would therapies targeted against CELF1 alleviate all aspects of DM1 pathogenesis. Nonetheless, partial therapies may improve quality of life for patients who currently have no treatment options.

## Acknowledgements

 We thank Natalie Vajda for helping maintain our mouse colony, Twishasri Dasgupta for helpful comments on the manuscript, and Yotam Blech-Hermoni for assistance with statistical analysis. DMPKS and DT960 plasmids were kindly provided by Dr. Thomas Cooper (Baylor College of Medicine). HSA^LR^ mice were generously provided by Dr. Charles Thornton (University of Rochester). 

## Funding information

This work was supported by a grant to A.N.L. from the Muscular Dystrophy Association (MDA87845). D.S.B. was supported by an NIH training grant (T32HL007914). 

## Competing interests

 The authors have declared that no competing interests exist.   
